# A Comparison of Airborne Microbial Load on Four Housed Dairy Farms

**DOI:** 10.3390/vetsci13040357

**Published:** 2026-04-05

**Authors:** Gergana Bachevska, Georgi Beev, Dimo Dimov, Elena Stancheva, Toncho Penev

**Affiliations:** 1 Department of Ecology and Animal hygiene, Faculty of Agriculture, Trakia University, 6000 Stara Zagora, Bulgaria; gergana.iotova@trakia-uni.bg (G.B.); dimo_1988@abv.bg (D.D.); elena.stancheva@trakia-uni.bg (E.S.); 2 Department of Biological Sciences, Faculty of Agriculture, Trakia University, Students Campus, 6000 Stara Zagora, Bulgaria; georgi.beev@trakia-uni.bg

**Keywords:** airborne microorganisms, dairy farms, bedding moisture, ventilation, microbial contamination

## Abstract

Airborne microbial contamination in dairy cattle housing is strongly influenced by housing conditions and management practices. This study evaluated the influence of environmental and housing parameters on total bacterial, coliform, and mold levels across four dairy farms. Microclimatic variables, including temperature, relative humidity, wind speed, bedding moisture, air volume per cow, particulate matter (PM_1_, PM_2.5_, PM_10_), and total volatile organic compounds (TVOCs), were measured. Comparative analyses showed that air volume per cow and bedding moisture were consistently associated with variability in total microbial and mold counts, while particulate matter and wind speed were linked to differences in airborne coliforms. Generalized linear mixed models indicated that most environmental variables did not have statistically significant effects, with the exception of farm type for coliforms and temperature for molds. The predominance of non-significant environmental effects, together with more consistent differences observed between farms, suggests that variability in airborne microbial levels is more strongly associated with farm-specific management and housing characteristics than with individual environmental parameters. Overall, the findings highlight the combined influence of housing design, management practices, and environmental conditions, emphasizing the importance of optimized ventilation and bedding management to improve air quality in dairy cattle housing.

## Simple Summary

This study investigated how environmental conditions, such as temperature, humidity, air movement, bedding moisture, and air space per cow, influence the levels of airborne microorganisms, including bacteria, coliforms, and molds, in different dairy farms. The results showed that some factors, particularly air volume per cow and bedding moisture, were associated with differences in microbial levels, while temperature played a key role in mold concentrations. However, overall, farm-specific characteristics and management practices appeared to have a greater influence than individual environmental parameters. These findings highlight the importance of proper housing design and management in maintaining good air quality and supporting animal housing in dairy production systems.

## 1. Introduction

Ambient air pollution is defined as the presence of harmful substances in the atmosphere, including gases, inorganic compounds and particulate matter, at concentrations that may adversely affect living organisms and environmental systems [1]. According to the World Health Organization [2], exposure to air pollutants represents a major environmental risk factor for human and animal health. In agricultural environments, workers involved in livestock production, such as dairy and beef cattle farming, may be exposed to airborne biological agents, representing a recognized occupational health risk [3]. Effective management of these biological hazards is essential to ensure both occupational safety and products’ hygiene. The European Union regulatory framework provides guidance on these issues, including Regulation (EC) No 178/2002 [4] establishing general principles and requirements of food law, Regulation (EC) No 852/2004 [5] on the hygiene of foodstuffs, Regulation (EC) No 853/2004 [6] laying down specific hygiene rules for food of animal origin, and Regulation (EU) 2017/625 [7] on official controls to verify compliance with food and feed law [4,5,6,7]. Incorporating these regulations highlights the importance of implementing biological safety measures in farming and food-processing environments to mitigate the risk posed by airborne hazards. The management of the second domain—physical environment—is directly and fundamentally linked to animal welfare, as housing conditions, including temperature, air quality, space allowance, and flooring, have a significant impact on both the physiological state and the subjective experiences of animals. Adverse environmental conditions may lead to discomfort, stress, and health problems, whereas well-managed environments support the expression of natural behaviors and promote positive affective states [8].

In this regard, dairy farms must implement management and production practices that safeguard both animal welfare and public health [9]. Within the European Union, the management of biological hazards in the food production chain is supported by preventive systems such as the Hazard Analysis and Critical Control Point approach, which aims to identify, evaluate, and control hazards that may compromise food safety [10]. The dairy farm environment harbors a diverse microbial community composed of ubiquitous bacteria, including microorganisms that are introduced and disseminated through interactions among animals, farm workers, and contaminated surfaces. This dynamic microbial ecosystem, if inadequately managed, can adversely affect the functioning and sustainability of dairy operations [11,12]. Zoonotic bacteria are of particular concern, as they are commonly associated with diseases such as pneumonia, salmonellosis, listeriosis, and mastitis in dairy cattle [13,14]. The bacterial agents responsible for these diseases, including *Salmonella* spp., *Listeria monocytogenes*, *Escherichia coli*, and *Staphylococcus aureus*, can adversely affect animal health and also pose a significant public health risk [15], as they may be excreted in feces, contaminate milk, and persist in the farm environment, thereby facilitating continuous transmission.

Effective monitoring and control of microbial contamination in cattle housing environments are crucial for ensuring proper animal welfare assessment [16,17].

This study aimed to quantify and compare airborne bacterial and fungal concentrations across four housed dairy farms to identify potential risk factors for microbial contamination in livestock environments.

## 2. Materials and Methods

### 2.1. Animals and Housing

The study encompassed four dairy farms with free housing and was conducted over a three-month period in autumn (September–November 2025, South Bulgaria). Specifically, the average milk yield for the studied farms were between 28 and 30 kg/day per cow. A detailed description of the housing conditions in each farm is provided in Table 1. Farm 1 was equipped with mechanical ventilation using fans, while the other three farms relied on natural ventilation. All buildings were open structures, with Farms 2 and 4 designed as shed-type housing.

### 2.2. Sampling Design

Airborne microorganisms were sampled on the four housed dairy farms using an Air Sampler Micro Biologique (model WA 98115) with 90 mm Petri dishes. A sampling time of 30 s was applied for total microbial and mold counts, while a 1 min sampling time was used for coliform counts, both at an airflow rate of 100 L/min. All samples were collected at a height of 1.2 m above the floor of the barns, corresponding approximately to the muzzle height of dairy cows. This sampling height was selected to avoid potential contamination of air samples from the bedding, as sampling at lower heights (e.g., 20–30 cm above the floor) may be influenced by direct emissions from the bedding material. Therefore, a height of 1.2 m was considered appropriate to obtain representative air samples within the animals’ breathing zone. At each farm, four to five sampling points were selected. The sampling points were selected to ensure adequate spatial distribution, being located at least 10 m from the beginning of the barn and at least 20 m apart from each other.

### 2.3. Microbial Analysis

The total number of airborne microorganisms was determined using nutrient agar (HiMedia, Mumbai, India). Coliform counts were assessed on Endo agar (HiMedia, Mumbai, India), while airborne molds were cultured on Sabouraud dextrose agar (HiMedia, Mumbai, India). After sampling, Petri dishes were transported to the laboratory in insulated cooling boxes with ice packs. Incubation conditions were as follows: Sabouraud dextrose agar at 22 °C for 72 h [18], and nutrient agar and Endo agar at 37 °C for 48 h [19].

### 2.4. Environmental Measurements

At each sampling point, air temperature, relative humidity, and air velocity were measured using a Kestrel 5400 device (Kestrel Instruments, Boothwyn, PA, USA), calibrated according to the manufacturer’s instructions. Fine particulate matter (PM1, PM2.5, and PM10) and total volatile organic compounds (TVOCs) were measured using a Testermeter WP6932 Smart Air Quality Detector (Guangzhou, China), calibrated according to the manufacturer’s specifications.

### 2.5. Bedding Analysis

At each sampling point, moisture content was determined from bedding and manure samples, whereas air measurements were conducted to assess environmental conditions. In Farms 1 and 3, five bedding samples were collected from each farm, while in Farms 2 and 4, four samples were collected per farm. All samples were taken from the top 0–5 cm of the bedding material to assess moisture content and its potential influence on airborne microbial concentrations. Bedding moisture was determined using a KERN DBS 60-3 moisture analyzer (Balingen, Germany) based on the thermogravimetric method [20]. In Farm 3, due to the absence of bedding material, manure was analyzed to determine its moisture content.

### 2.6. Data Standardization

All air sample results were adjusted to account for differences in sampling time and airflow rate, ensuring comparability across all farms. Specifically, data were standardized to a uniform sampling volume of 100 L to allow direct comparison of microbial concentrations between sampling points and farms.

### 2.7. Statistical Analysis

The normality of the transformed data was assessed using the Shapiro–Wilk test. The results of the normality tests are provided in the supplementary file. As most of the data remained non-normally distributed, a non-parametric approach was applied. Differences among groups were analyzed using the Kruskal–Wallis test. Statistical analyses were performed using GraphPad Prism, version 10.6.1. (GraphPad Software, Inc., La Jolla, CA, USA).

Airborne microbial concentrations, including total microbial counts, coliform bacteria, and molds, were statistically analyzed to determine the influence of farm characteristics and environmental parameters in dairy farms. Because the response variables represented count data with potential overdispersion, generalized linear mixed models (GLMMs) with a negative binomial distribution and log link function were applied.

The dependent variables included total airborne microbial counts, airborne coliform counts, and airborne mold counts. The explanatory variables consisted of farm type and location as categorical fixed effects, while environmental parameters were included as continuous predictors. These environmental variables comprised relative humidity (%), air temperature (°C), wind speed (m/s), bedding humidity (%), air volume per cow (m^3^), particulate matter concentrations (PM1, PM2.5, PM10), and total volatile organic compounds (TVOCs).

For categorical variables, reference categories were defined to allow comparisons among groups. Model outputs included regression coefficients (B), standard errors (SE), exponentiated coefficients (Exp(B)), 95% confidence intervals (CI), Wald χ^2^ statistics, degrees of freedom (df), and *p*-values, which were used to evaluate the strength and significance of associations between predictors and airborne microbial concentrations.

The model included Farm Type and Location as categorical predictors and environmental variables—RH%, Temperature, Wind Speed, Bedding Humidity, Air Volume per Cow, PM1, PM2.5, PM10, and TVOCs—as continuous covariates.

The Exp(B) values indicate the rate ratio associated with a one-unit change in the predictor, with reference categories set for Farm Type (Farm 4) and Location (Location 2).

The GLMM analysis was performed using IBM SPSS Statistics version 26 (IBM Corp., Armonk, NY, USA). A *p*-value < 0.05 was considered statistically significant.

The effect size of differences in airborne microbial load between farms was calculated using the following formula:

$$r=\frac{z}{\surd N}$$

where z is the standardized test statistic derived from the non-parametric analysis and N is the total number of observations.

## 3. Results

### 3.1. Microclimatic Conditions Across Farms

Microclimatic parameters differed among the four farms (Table 2). Relative humidity ranged from 64.73 ± 7.76% (Farm 4) to 71.07 ± 10.89% (Farm 1). Bedding moisture showed substantial variation, with the lowest values in Farm 1 (31.75 ± 11.14%) and the highest in Farm 3 (84.09 ± 1.85%). Air velocity was greatest in Farm 1 (1.01 ± 0.77 m/s) and lowest in Farm 3 (0.44 ± 0.55 m/s). Marked differences were also observed in air volume per cow, ranging from 32.62 ± 9.16 m^3^/cow (Farm 2) to 236.3 ± 0.57 m^3^/cow (Farm 4).

### 3.2. Differences in Environmental Parameters in Relation to Microbial Groups

The Kruskal–Wallis test revealed significant differences between farms for several environmental variables in relation to microbial groups. For total bacterial counts (TBC), significant effects were observed for bedding moisture (H = 10.42, *p* = 0.015) and air volume per cow (H = 35.29, *p* < 0.001). In the case of coliforms, relative humidity (H = 8.369, *p* = 0.039), particulate matter concentrations (PM_1_: H = 12.27, *p* = 0.006; PM_2.5_: H = 11.74, *p* = 0.008; PM_10_: H = 12.21, *p* = 0.007), air volume per cow (H = 23.38, *p* < 0.001), and wind speed (H = 15.17, *p* = 0.002) showed statistically significant differences. For molds, both bedding moisture (H = 23.43, *p* < 0.001) and air volume per cow (H = 34.88, *p* < 0.001) were significantly associated with differences between farms (Table 3).

**Table 3:** Summary of significant associations (Kruskal–Wallis H, *p*-values).

Variable	Microbial Group	H	df	*p*
Bedding Moisture	TBC	10.42	3	0.015
Volume per cow	TBC	35.29	3	<0.001
RH	Coliforms	8.369	3	0.039
PM1	Coliforms	12.27	3	0.006
PM2.5	Coliforms	11.74	3	0.008
PM10	Coliforms	12.21	3	0.007
Volume per cow	Coliforms	23.38	3	<0.001
Wind Speed	Coliforms	15.17	3	0.002
Bedding Moisture	Molds	23.43	3	<0.001
Volume per cow	Molds	34.88	3	<0.001

Only selected significant variables are shown; complete results for all measured parameters are provided in the Supplementary Table S4.

### 3.3. Comparative Analysis of Environmental Effects on Airborne Microbial Counts

Comparative analysis revealed moderate-to-large differences in total airborne microbial counts between farms across several environmental parameters (Table 4). Bedding moisture showed consistently large effects in comparisons involving Farm 1 (vs. Farms 2, 3, and 4; r = 0.46–0.52). Wind speed demonstrated a large effect between Farms 1 and 3 (r = 0.48) and a moderate effect between Farms 2 and 3 (r = 0.35). Air volume per cow exhibited the strongest differences, with large effect sizes across all comparisons (r = 0.72–0.88), particularly between Farms 2, 3, and 4. Temperature differences were moderate, observed between Farms 1 and 3 (r = 0.40) and Farms 2 and 3 (r = 0.33). Overall, air volume per cow and bedding moisture emerged as the most influential factors associated with variability in airborne microbial counts among farms.

### 3.4. Comparative Analysis of Environmental Effects on Airborne Coliform Counts

Comparative analysis demonstrated moderate-to-large environmental effects on airborne coliform counts across farms (Table 5). Bedding humidity showed consistently large effects in comparisons involving Farm 1 (vs. Farms 2, 3, and 4; r = 0.46–0.52). Wind speed exhibited predominantly large effects, particularly in comparisons involving Farm 1 (r = 0.51–0.69), although a moderate effect was observed between Farms 2 and 3 (r = 0.35). Air volume per cow again showed the strongest and most consistent differences, with large effect sizes across all comparisons (r = 0.72–0.88). Particulate matter also contributed to variability in coliform counts. PM_1_ demonstrated large effects across multiple farm comparisons (r = 0.72–0.88), while PM_10_ showed both moderate and large effects (r = 0.31–0.67). Total volatile organic compounds (TVOCs) exhibited large effects in comparisons involving Farm 1 (r = 0.42–0.47). Temperature differences were moderate, observed between Farms 1 and 3 (r = 0.40) and Farms 2 and 3 (r = 0.33). Overall, air volume per cow, particulate matter (PM_1_), and wind speed emerged as the most influential environmental factors associated with variation in airborne coliform counts among farms.

### 3.5. Comparative Analysis of Environmental Effects on Airborne Mold Counts

Comparative analysis indicated moderate-to-large differences in airborne mold counts between farms across several environmental parameters (Table 6). Bedding humidity showed both moderate and large effects, with the strongest difference observed between Farms 1 and 3 (r = 0.89), while additional large effects were identified between Farms 1 and 4 and Farms 2 and 3 (r = 0.50–0.52). Moderate effects were also observed between Farms 1 and 2 and Farms 3 and 4 (r = 0.37–0.38). Wind speed demonstrated only moderate effects, limited to comparisons involving Farm 1 (vs. Farms 2 and 3; r = 0.33–0.34). In contrast, air volume per cow exhibited consistently large effects across all comparisons (r = 0.61–0.99), with the highest effect observed between Farms 2 and 4 (r = 0.99). Overall, air volume per cow and bedding humidity emerged as the primary factors associated with variability in airborne mold counts among farms, while wind speed showed a comparatively weaker influence.

### 3.6. GLMM Analysis of Factors Affecting Airborne Microbial Counts

The generalized linear mixed model (GLMM) with a negative binomial distribution was used to evaluate the effects of farm type, location, and environmental parameters on total airborne microbial counts across dairy farms (Table 7). None of the predictors had statistically significant effects on total airborne microbial counts (*p* > 0.05). The 95% Wald confidence intervals for the coefficients included zero, further confirming the lack of significant effects. Specifically, changes in relative humidity (RH%) and temperature were associated with small changes in microbial counts (Exp(B) = 0.99 and 1.06, respectively), whereas air pollution indicators (PM1, PM2.5, PM10, TVOCs) and housing parameters (wind speed, bedding humidity, air volume per cow) showed minimal influence on microbial levels.

### 3.7. GLMM Analysis of Factors Affecting Airborne Coliform Counts

The effects of farm type, location, and environmental parameters on airborne coliform counts in dairy farms were evaluated using generalized linear mixed models (GLMM) with a negative binomial distribution (Table 8).

The model indicated that Farm Type had the strongest effect on airborne coliform counts, with Farms 1, 2, and 3 showing significantly lower counts compared to the reference farm (Farm 4), as indicated by Exp(B) values of 0.12, 0.35, and 0.23, respectively (*p* < 0.01). No significant differences were detected for Location or for any of the continuous environmental variables (*p* > 0.05), although bedding humidity (*p* = 0.069), PM2.5 (*p* = 0.079), PM10 (*p* = 0.087), and TVOCs (*p* = 0.072) showed trends toward influencing coliform counts.

### 3.8. GLMM Analysis of Factors Affecting Airborne Mold Counts

The effects of farm type, location, and environmental parameters on airborne mold counts in dairy farms were analyzed using generalized linear mixed models (GLMM) with a negative binomial distribution.

As shown in Table 9, Temperature was the only factor significantly associated with airborne mold counts (*p* = 0.034), with higher temperatures corresponding to slightly increased mold levels (Exp(B) = 1.09). None of the other environmental variables or categorical factors (Farm Type and Location) showed statistically significant effects (*p* > 0.05), although minor trends were observed for some variables.

These results suggest that ambient temperature may influence airborne mold concentrations, whereas farm-specific characteristics and other measured environmental parameters had no detectable impact under the conditions of the studied farms.

## 4. Discussion

The results indicate that particulate matter fractions (PM1, PM2.5, and PM10), bedding moisture, wind speed, and available barn volume per cow exert the strongest influence on the total counts of airborne microorganisms, coliforms, and molds (Table 3). Similar findings have been reported in studies conducted on pig farms, where Tang et al. [21] demonstrated that increasing PM2.5 concentrations were associated with higher airborne microbial loads, particularly molds. In contrast, the present investigation assessed not only particulate matter but also housing-related parameters, including bedding moisture and building volume per animal. The results of the Kruskal–Wallis test indicate that bedding moisture and barn volume per cow are the most influential factors affecting the total microbial load across the studied farms (Table 4, Table 5 and Table 6). This highlights the critical role of microclimatic conditions and stocking density in shaping airborne microbial contamination in dairy housing systems. According to Callejo [22], an optimal space allowance of 35–40 m^3^ per cow is recommended to prevent excessive concentrations of mesophilic bacteria, referred to in the present study as total bacterial count (TBC). Data presented in Table 2 show that Farm 2 provided the lowest volume per cow (32.62 m^3^/cow), which is below the recommended range. Farm 3 followed with 48.28 m^3^/cow, whereas Farms 1 and 4 offered substantially larger volumes per cow, amounting to 157.5 and 236.3 m^3^/cow, respectively. These differences in space availability likely contribute to the observed variability in airborne microbial concentrations among the farms. Therefore, the volume of space available per cow may play a key role in animal welfare by influencing the microbial load of the indoor air. The comparative analysis between the studied farms showed that the most pronounced differences in the total number of airborne microorganisms were associated with the available volume per cow (Table 4). According to the Kruskal–Wallis test, the differences in measured parameters between farms ranged from 0.72 to 0.88, with the effect sizes (r) indicating substantial variation among farms.

Bedding moisture is another factor that strongly influences housing conditions, as reflected by the total microbial load in the indoor air of the studied farms. Three different bedding systems were identified across these farms. Farm 1 utilized a deep, non-replaceable bedding system, whereas in Farms 2 and 4 cows were housed on deep bedding with periodic straw additions, applied according to the degree of bedding contamination. In contrast, Farm 3 employed rubber mattresses in individual cubicles. Previous studies have demonstrated that airborne microbial concentrations in dairy barns increase when bedding material is not adequately cleaned or replaced at sufficient frequency [23,24]. Additionally, Monsallier et al. [25] reported that the use of straw as bedding material may lead to elevated levels of total airborne bacteria. In the present study, two of the investigated farms (farms 2 and 4) used straw-based bedding systems, which necessitates a more detailed comparative analysis to assess the role of bedding moisture as a determinant of airborne microbial contamination and, consequently, dairy cow housing conditions.

With respect to airborne coliform concentrations, the present results indicate that several environmental and housing-related parameters play a significant role. As shown in Table 5, statistically significant differences between farms were associated with bedding moisture, wind speed, barn air volume per cow, temperature, particulate matter fractions (PM1 and PM10), and TVOCs, with effect sizes (r) ranging from moderate to large. In this study, the variation in space allowance per cow was considerable, ranging from 8.06 to 59.73 m^2^/cow (Table 1). This wide range necessitates a farm-specific analysis of airborne coliform and mold concentrations in relation to relative humidity. Organic dust particles (PM1, PM2.5, and PM10) originate primarily from manure, feed, and animal surfaces and may act as carriers of bacteria, including coliforms. Previous research in dairy farms has shown that increased bioaerosol concentrations are associated with environmental conditions that also influence particulate matter levels, such as ventilation and housing management [19]. Previous studies have demonstrated that both housing volume per animal and bedding moisture are key determinants of airborne microbial contamination in dairy farms. Reduced air space per cow has been associated with increased concentrations of airborne microorganisms, due to limited dilution and ventilation capacity [26]. Similarly, higher moisture levels in bedding create favorable conditions for microbial growth, as humidity supports bacterial proliferation and the decomposition of organic material [26]. In addition, lower dry matter content in bedding has been linked to increased bacterial counts, indicating that moist bedding represents an important source of microorganisms [27].

In the study by Kic [28], particulate matter in cattle barns was shown to originate primarily from bedding materials and housing design, with lower dust concentrations observed in open-type barns. In the present study, all investigated farms were of open housing type, which may have contributed to reduced overall particulate levels. Paduch et al. [29] demonstrated that bedding materials can serve as a significant reservoir of bacteria, including coliforms, many of which are pathogenic and capable of causing diseases in dairy cows. Similarly, Murphy et al. [30] reported that bedding may act as a source of spore-forming bacteria, influencing the microbial load of the surrounding environment and contributing to the subsequent contamination of raw milk. Taken together, these findings indicate that both the type of bedding material and its management practices play a critical role in shaping airborne microbial contamination and represent key determinants of dairy cow housing conditions.

Airborne mold concentrations are influenced by environmental conditions, particularly bedding moisture, as increased humidity in the bedding promotes fungal growth and contributes to higher levels of molds in the air [26]. When compared with the optimal space recommendation of 35–40 m^3^ per cow proposed by Callejo [22], Farm 1 (157.6 m^3^ per cow) and Farm 4 (236.3 m^3^ per cow) both substantially exceeded these values. In contrast, Farm 2 (32.62 m^3^ per cow) and Farm 3 (48.29 m^3^ per cow) were closer to the optimal range suggested by the aforementioned author. The effect size for the difference between Farm 1 and Farm 2 was 0.82, whereas the effect size between Farm 2 and Farm 4 was 0.99. Previous studies have demonstrated that barn space availability per animal can influence multiple microclimatic parameters, including increases in pathogenic microorganism concentrations [31,32]. Other factors such as animal activity, ventilation, bedding type, and floor characteristics have also been shown to affect various airborne contaminants, including bacterial loads [33].

Several studies have demonstrated that airborne microbial concentrations are influenced by environmental conditions such as air temperature and relative humidity. For example, temperature and humidity were shown to jointly affect mold and bacterial levels in indoor environments [34], and seasonal increases in airborne bacteria were associated with higher temperatures and humidity in livestock housing [35]. Heat events and fluctuations in humidity have also been reported to alter airborne bacterial community composition [36]. In the present study, however, the generalized linear mixed model indicated that these environmental factors had limited impact on total airborne microbial counts (Table 7). The Exp(B) values, with reference categories set for Farm Type (Farm 4) and Location (Location 2), showed only small changes in microbial counts for relative humidity (0.99) and temperature (1.06), while air pollution indicators (PM1, PM2.5, PM10, TVOCs) and housing parameters (wind speed, bedding humidity, air volume per cow) had minimal influence. These results suggest that, under the conditions of the studied farms, overall airborne microbial concentrations are likely shaped by a combination of management practices, including bedding type, wind speed, air volume per cow, ambient temperature, and air relative humidity, rather than by any single environmental factor.

The results from Table 8 indicate that farm type has the strongest influence on airborne coliform counts, with Farms 1, 2, and 3 showing significantly lower levels compared to the reference farm (Farm 4). In contrast, location and the measured environmental parameters, including relative humidity, temperature, wind speed, bedding humidity, air volume per cow, particulate matter, and TVOCs, did not have significant effects, although some trends were observed for bedding humidity and air pollutants. It is well established that bedding type can influence the nature and level of microbial air contamination in dairy barns [26]. The higher airborne coliform concentrations are likely associated with the use of straw bedding, which has been reported to contain more than 10^6^ colony-forming units (CFU) per gram [37]. These findings suggest that farm-specific management practices and characteristics, rather than general environmental conditions, are likely the main determinants of airborne coliform concentrations.

The analysis of airborne mold counts (Table 9) indicated that temperature was the only factor significantly associated with mold levels, with higher temperatures corresponding to slightly increased counts (Exp(B) = 1.09, *p* = 0.034). None of the other factors, including farm type, location, relative humidity, wind speed, bedding humidity, air volume per cow, or air pollutants (PM1, PM2.5, PM10, TVOCs), showed statistically significant effects, although minor trends were observed for some variables. These findings suggest that, under the conditions of the studied farms, temperature is the main determinant of airborne mold concentrations, while other environmental and housing parameters may contribute under different conditions. Clean air is generally not a suitable environment for microbial growth due to limited nutrients [38], and microorganisms can travel in the air attached to dust particles or droplets [39], which may help explain the presence of mold in farm air. Overall, the results highlight the combined influence of environmental factors and microbial dispersal mechanisms on airborne mold levels in dairy farms.

## 5. Limitations

Despite providing valuable insights into the relationships between environmental and housing-related parameters and airborne microbial concentrations in dairy farms, the present study has several limitations that should be acknowledged. First, the study was conducted on only four farms, each with different housing systems and management practices. This variability, combined with the limited sample size, may restrict the generalizability of the findings to other production systems and conditions. Second, the study was carried out within a single season over a three-month period, which does not allow for the assessment of seasonal variability in environmental conditions and airborne microbial concentrations. Third, in order to investigate the relationships between airborne total bacteria, coliforms, and molds and environmental conditions, a selected set of environmental parameters was included in the analysis. However, other potentially relevant factors, such as ventilation rate, animal activity, and other management practices, were not directly measured and may have influenced the results. Finally, the microbiological analysis was limited to general indicators, including total microorganisms, coliforms, and molds, without detailed identification at the species level. This limits the ability to assess the presence of specific microorganisms and their potential health implications.

## 6. Conclusions

This study demonstrated that microclimatic conditions and housing parameters vary considerably among dairy farms and are associated with differences in airborne microbial loads. Among the evaluated factors, air volume per cow and bedding moisture consistently showed strong associations with variability in total microbial counts and molds, while particulate matter and wind speed were more closely related to airborne coliform levels. The generalized linear mixed model revealed that most environmental parameters had no statistically significant effect on total microbial and coliform counts, although certain variables showed trends, suggesting potential indirect or context-dependent influences. In contrast, farm type emerged as a key determinant of airborne coliform concentrations, indicating the importance of farm-specific management practices. For airborne molds, temperature was the only significant predictor, suggesting that thermal conditions may play a critical role in molds proliferation and dispersion in dairy housing environments. Overall, the findings indicate that airborne microbial contamination is influenced by a complex interaction of environmental and management-related factors, with farm characteristics playing a more prominent role than individual microclimatic parameters. These results highlight the importance of optimizing housing design, ventilation, and bedding management to improve air quality and support animal health and welfare in dairy farms.

## Figures and Tables

**Table 1:** Housing conditions of the studied farms.

Farms	Housing System	Type of Bedding	Space per Cow (m^2^/Cow) $\bar{x}$ ± SD
Farm 1	Loose housing with permanent deep litter	Deep litter housing system	20.92 ± 0.86
Farm 2	Loose housing on deep bedding	Deep bedding with periodic addition of straw	10.04 ± 0.43
Farm 3	Free-stall housing with individual cubicles	Cubicles fitted with rubber mats	8.06 ± 0.32
Farm 4	Loose housing on deep bedding	Deep bedding with periodic addition of straw	59.73 ± 2.97

**Table 2:** Environmental and housing parameters of the studied dairy farms.

Farm	Relative Humidity, % Mean ± SD	Bedding Moisture, % Mean ± SD	Wind Speed, m/s Mean ± SD	Volume/Cow, m^3^/Cow Mean ± SD
Farm 1 (*n* = 15)	71.07 ± 10.89	31.75 ± 11.14	1.01 ± 0.77	157.6 ± 0.90
Farm 2 (*n* = 12)	64.88 ± 12.67	54.03 ± 21.22	0.53 ± 0.31	32.62 ± 9.16
Farm 3 (*n* = 15)	65.66 ± 11.80	84.09 ± 1.85 *	0.44 ± 0.55	48.29 ± 1.89
Farm 4 (*n* = 12)	64.73 ± 7.76	65.08 ± 13.37	0.61 ± 0.41	236.3 ± 0.57

Note: * Value refers to manure, not bedding material.

**Table 4:** Moderate-to-large pairwise differences in total airborne microbial counts among dairy farms across environmental parameters.

Comparison (Farm vs. Farm)	Factor/Environmental Parameter	Z	N	Effect Size r	Interpretation
Farm 1–Farm 2	Bedding humidity	2.651	26	0.52	Large
Farm 1–Farm 3	Bedding humidity	2.780	29	0.52	Large
Farm 1–Farm 4	Bedding humidity	2.322	26	0.46	Large
Farm 1–Farm 3	Wind speed	2.629	30	0.48	Large
Farm 2–Farm 3	Wind speed	1.855	28	0.35	Moderate
Farm 1–Farm 2	Air volume per cow	3.668	26	0.72	Large
Farm 1–Farm 3	Air volume per cow	4.012	30	0.73	Large
Farm 2–Farm 4	Air volume per cow	4.308	24	0.88	Large
Farm 3–Farm 4	Air volume per cow	4.671	28	0.88	Large
Farm 1–Farm 3	Temperature	2.134	28	0.40	Moderate
Farm 2–Farm 3	Temperature	1.772	28	0.33	Moderate

Notes: Effect size (r) was calculated as r = Z/√N. Interpretation followed Cohen’s thresholds: 0.10 = small, 0.30 = moderate, and 0.50 = large. N represents the total number of observations in each comparison.

**Table 5:** Moderate-to-large environmental effects on airborne coliform counts across dairy farms.

Comparison (Farm vs. Farm)	Factor/Environmental Parameter	Z	N	Effect Size r	Interpretation
Farm 1–Farm 2	Bedding humidity	2.651	26	0.52	Large
Farm 1–Farm 3	Bedding humidity	2.780	29	0.52	Large
Farm 1–Farm 4	Bedding humidity	2.322	26	0.46	Large
Farm 1–Farm 2	Wind speed	2.940	26	0.58	Large
Farm 1–Farm 4	Wind speed	3.532	26	0.69	Large
Farm 1–Farm 3	Wind speed	2.629	30	0.48	Large
Farm 2–Farm 3	Wind speed	1.855	28	0.35	Moderate
Farm 1–Farm 2	Air volume per cow	3.668	26	0.72	Large
Farm 1–Farm 3	Air volume per cow	4.012	30	0.73	Large
Farm 2–Farm 4	Air volume per cow	4.308	24	0.88	Large
Farm 3–Farm 4	Air volume per cow	4.671	28	0.88	Large
Farm 1–Farm 3	Temperature	2.134	28	0.40	Moderate
Farm 2–Farm 3	Temperature	1.772	28	0.33	Moderate
Farm 1–Farm 2	PM1	3.668	26	0.72	Large
Farm 1–Farm 3	PM1	4.012	30	0.73	Large
Farm 1–Farm 4	PM1	4.308	26	0.88	Large
Farm 3–Farm 4	PM1	4.671	28	0.88	Large
Farm 1–Farm 2	PM10	2.250	26	0.44	Large
Farm 1–Farm 3	PM10	1.960	30	0.36	Moderate
Farm 1–Farm 4	PM10	3.410	26	0.67	Large
Farm 3–Farm 4	PM10	1.514	28	0.31	Moderate
Farm 1–Farm 2	TVOCs	2.395	26	0.47	Large
Farm 1–Farm 4	TVOCs	2.139	26	0.42	Large

Notes: Effect size (r) was calculated as r = Z/√N. Interpretation followed Cohen’s thresh-olds: 0.10 = small, 0.30 = moderate, and 0.50 = large. N represents the total number of observations in each comparison.

**Table 6:** Moderate-to-large effects of environmental parameters on airborne mold counts across dairy farms.

Comparison (Farm vs. Farm)	Factor/Environmental Parameter	Z	N	Effect Size r	Interpretation
Farm 1–Farm 2	Bedding humidity	1.952	26	0.38	Moderate
Farm 1–Farm 3	Bedding humidity	4.793	29	0.89	Large
Farm 1–Farm 4	Bedding humidity	2.645	26	0.52	Large
Farm 2–Farm 3	Bedding humidity	2.617	27	0.50	Large
Farm 3–Farm 4	Bedding humidity	1.913	27	0.37	Moderate
Farm 1–Farm 2	Wind Speed	1.740	26	0.34	Moderate
Farm 1–Farm 3	Wind Speed	1.811	30	0.33	Moderate
Farm 1–Farm 2	Air Volume per Cow	4.180	26	0.82	Large
Farm 1–Farm 3	Air Volume per Cow	3.355	30	0.61	Large
Farm 2–Farm 4	Air Volume per Cow	4.860	24	0.99	Large
Farm 3–Farm 4	Air Volume per Cow	4.104	28	0.78	Large

Notes: Effect size (r) was calculated as r = Z/√N. Interpretation followed Cohen’s thresholds: 0.10 = small, 0.30 = moderate, and 0.50 = large. N represents the total number of observations in each comparison.

**Table 7:** GLMM results for total airborne microbial counts in dairy farms (Negative binomial distribution).

Factor	Level/Category	B	SE	Exp(B)	95% CI (B)	Wald χ^2^	df	*p*-Value
Farm Type	1	−0.299	0.361	0.74	−0.818–0.597	0.094	1	0.759
	2	−0.458	0.368	0.63	−1.120–0.321	1.180	1	0.277
	3	−0.048	0.353	0.95	−0.740–0.644	0.018	1	0.893
	4 (ref)	0	–	1.00	–	–	–	–
Location	1	0.471	0.336	1.60	−0.187–1.128	1.968	1	0.161
	2 (ref)	0	–	1.00	–	–	–	–
RH%	–	−0.015	0.012	0.99	−0.040–0.009	1.559	1	0.212
Temperature	–	0.056	0.042	1.06	−0.026–0.138	1.772	1	0.183
Wind Speed	–	−0.154	0.248	0.86	−0.639–0.332	0.384	1	0.535
Bedding Humidity	–	−0.009	0.011	0.99	−0.030–0.012	0.679	1	0.410
Air Volume per Cow	–	−0.001	0.032	1.00	−0.064–0.062	0.001	1	0.978
PM1	–	0.083	0.069	1.09	−0.053–0.219	1.416	1	0.234
PM2.5	–	0.072	0.056	1.08	−0.038–0.182	1.659	1	0.198
PM10	–	0.058	0.047	1.06	−0.034–0.150	1.535	1	0.215
TVOCs	–	0.373	0.907	1.45	−1.404–2.151	0.169	1	0.681

Notes: Exp(B) = rate ratio for a one-unit increase in the predictor; Ref = reference category. SE = standard error; CI = 95% Wald confidence interval; *p*-value from Wald χ^2^ test. Models are Negative Binomial GLMM.

**Table 8:** GLMM results for airborne coliform counts in dairy farms (Negative binomial distribution).

Factor	Level/Category	B	SE	Exp(B)	95% CI (B)	Wald χ^2^	df	*p*-Value
Farm Type	1	−2.258	0.384	0.12	−3.047–−1.474	30.52	1	**<0.001**
	2	−1.070	0.387	0.35	−1.831–−0.312	6.98	1	**0.009**
	3	−1.482	0.359	0.23	−2.301–−0.755	16.05	1	**<0.001**
	4 (ref)	0	–	1.00	–	–	–	–
Location	1	0.228	0.341	1.27	−0.546–1.018	0.55	1	0.528
	2 (ref)	0	–	1.00	–	–	–	–
Relative Humidity (%)	–	0.014	0.015	1.01	−0.014–0.042	0.931	1	0.335
Temperature	–	−0.040	0.042	0.96	−0.123–0.043	0.885	1	0.347
Wind Speed	–	0.150	0.290	1.16	−0.419–0.719	0.268	1	0.605
Bedding Humidity	–	−0.021	0.011	0.98	−0.043–0.002	3.310	1	0.069
Air Volume per Cow	–	0.013	0.034	1.01	−0.053–0.079	0.150	1	0.699
PM1	–	0.114	0.074	1.12	−0.031–0.258	2.389	1	0.122
PM2.5	–	0.106	0.061	1.11	−0.012–0.225	3.087	1	0.079
PM10	–	0.086	0.050	1.09	−0.013–0.185	2.924	1	0.087
TVOCs	–	−1.912	1.063	0.15	−3.995–0.172	3.234	1	0.072

Notes: Exp(B) = rate ratio for a one-unit increase in the predictor; Ref = reference category. SE = standard error; CI = 95% Wald confidence interval; *p*-value from Wald χ^2^ test. Models are Negative Binomial GLMM; significant effects (*p* < 0.05) would be shown in bold.

**Table 9:** GLMM results for airborne mold counts in dairy farms (Negative binomial distribution).

Factor	Level/ Category	B	SE	Exp(B)	95% CI (B)	Wald χ^2^	df	*p*-Value
Farm Type	1	−0.843	0.369	0.43	−0.858–0.589	0.133	1	0.715
	2	−0.074	0.371	0.93	−0.773–0.681	0.015	1	0.903
	3	−0.265	0.349	0.77	−0.939–0.427	0.540	1	0.463
	4 (ref)	0	–	1.00	–	–	–	–
Location	1	0.113	0.324	1.12	−0.524–0.744	0.115	1	0.734
	2 (ref)	0	–	1.00	–	–	–	–
Relative Humidity (%)	–	−0.017	0.013	0.98	−0.042–0.009	1.672	1	0.196
Temperature	–	0.085	0.040	1.09	0.006–0.164	4.473	1	**0.034**
Wind Speed	–	−0.017	0.243	0.98	−0.494–0.460	0.005	1	0.944
Bedding Humidity	–	−0.002	0.011	1.00	−0.022–0.019	0.020	1	0.887
Air Volume per Cow	–	−0.020	0.031	0.98	−0.081–0.042	0.399	1	0.527
PM1	–	0.010	0.067	1.01	−0.121–0.142	0.025	1	0.876
PM2.5	–	0.009	0.055	1.01	−0.098–0.116	0.027	1	0.870
PM10	–	0.008	0.046	1.01	−0.081–0.098	0.034	1	0.854
TVOCs	–	0.153	0.947	1.17	−1.703–2.010	0.026	1	0.871

Notes: Exp(B) = rate ratio for a one-unit increase in the predictor; Ref = reference category. SE = standard error; CI = 95% Wald confidence interval; *p*-value from Wald χ^2^ test. Models are Negative Binomial GLMM; significant effects (*p* < 0.05) would be shown in bold.

## Data Availability

The original contributions presented in this study are included in the article/supplementary material. Further inquiries can be directed to the corresponding author.

## Supplementary Materials

The following supporting information can be downloaded at: https://www.mdpi.com/article/10.3390/vetsci13040357/s1, Table S1. Results of the Shapiro–Wilk test for normality of log_10_-transformed total bacterial counts (TBC) in the studied farms; Table S2. Results of the Shapiro–Wilk test for normality of log_10_-transformed coliform counts in the studied farms; Table S3. Results of the Shapiro–Wilk test for normality of log_10_-transformed Molds in the studied farms; Table S4. Results of the Kruskal–Wallis test for the studied variables.
