# Supplementary material for: A Comparison of Airborne Microbial Load on Four Housed Dairy Farms
Source: Vet Sci. 2026 Apr 5;13(4):357. doi: 10.3390/vetsci13040357 (PMC13120651; doi:10.3390/vetsci13040357)
Supplement: Supplementary file 1 [file vetsci-13-00357-s001.zip › vetsci-4185420-supplementary.pdf]

Table S1. Results of the Shapiro–Wilk test for normality of log_{10}-transformed total bacterial counts (TBC) in the studied farms

Variable	Farm 1 (W / p/ Yes-No)	Farm 2 (W / p/ Yes-No)	Farm 3 (W / p/ Yes-No)	Farm 4 (W / p/ Yes-No)
TBC log_{10}(CFU+1) (n=15)	0.8797/0.31*/No	0.7501/<0.001***/No	0.8145/0.004**/No	0.9497/0.520^{ns}/ Yes
TBC log_{10}(CFU+1)/RH (n=15)	0.9640/0.708^{ns}/Yes	0.9166/0.171^{ns}/Yes	0.8435/0.011*/No	0.9315/0.287^{ns}/Yes
TBC log_{10}(CFU+1)/PM1 (n=15)	0.9018/0.073^{ns}/Yes	0.7778/0.002**/No	0.8068/0.003**/No	0.8963/0.084^{ns}/Yes
TBC log_{10}(CFU+1)/PM2.5 (n=15)	0.9579/0.592^{ns}/Yes	0.7807/0.002**/No	0.8491/0.013*/No	0.9917/>0.999^{ns}/Yes
TBC log_{10}(CFU+1)/PM10 (n=15)	0.9440/0.3691^{ns}/Yes	0.7806/0.002**/No	0.8472/0.012*/No	0.9896/>.999^{ns}/Yes
TBC log_{10}(CFU+1)/TVOCs (n=15)	0.9485/0,4338$^{ns/}$Yes	0.9646/0,77^{ns}/Yes	0.9032/0.091^{ns}/Yes	0.9016/0.101^{ns}/Yes
TBC log_{10}(CFU+1)/BM (n=15)	0.9039/0.1285^{ns}/Yes	0.8648/0.056^{ns}/Yes	0.8092/0.005**/No	0.9301/0.381^{ns}/Yes
TBC log_{10}(CFU+1)/WS (n=15)	0.9289/0.2085^{ns}/Yes	0.7597/0.001**/No	0.8777/0.036*/No	0.8488/0.017*/No
TBC log_{10}(CFU+1)/V/cow (n=15)	0.3509/<0,0001****/No	0.7876/0.006**/No	0.9333/0.274^{ns}/Yes	0.9024/0.170^{ns}/Yes

Notes: Values are presented as Shapiro–Wilk W statistic, p-value, and normality decision (Yes/No). Normal distribution was assumed at p > 0.05. RH—relative humidity; PM – particle matters; TVOCs – Total Volatile Organic Compounds; BM – Bedding Moisture; WS – Wind Speed; V/cow – volume per cow.

Table S2. Results of the Shapiro–Wilk test for normality of log_{10}-transformed coliform counts in the studied farms

Variable	Farm 1 (W / p/ Yes-No)	Farm 2 (W / p/ Yes-No)	Farm 3 (W / p/ Yes-No)	Farm 4 (W / p/ Yes-No)
Coliforms log_{10}(CFU+1) (n=15)	0.9344/0.2577^{ns}/Yes	0.7501/<0.001***/No	0.9647/0.747^{ns}/Yes	0.8633/0.027*/No
Coliforms log_{10}(CFU+1)/RH (n=15)	0.9584/0.6013^{ns}/Yes	0.9434/0.4274^{ns}/Yes	0.9291/0.2646^{ns}/Yes	0.8656/0.0364*/No
Coliforms log_{10}(CFU+1)/PM1 (n=15)	0.9322/0.2370^{ns}/Yes	0.8675/0.0310*/No	0.8633/0.0269*/No	0.7974/0.0046**/No
Coliforms log_{10}(CFU+1)/PM2.5 (n=15)	0.9279/0.2005^{ns}/Yes	0.8851/0.0566^{ns}/Yes	0.8847/0.0559^{ns}/Yes	0.8873/0.0739^{ns}/Yes
Coliforms log_{10}(CFU+1)/PM10 (n=15)	0.8943/0.0547^{ns}/Yes	0.8802/0.0478*/No	0.8931/0.0746^{ns}/Yes	0.8852/0.0690^{ns}/Yes
Coliforms log_{10}(CFU+1)/TVOCs (n=15)	0.9335/0.2487^{ns}/Yes	0.9807/0.9742^{ns}/Yes	0.9591/0.6767^{ns}/Yes	0.9319/0.3244^{ns}/Yes
Coliforms log_{10}(CFU+1)/Hbed (n=12)	0.8952/0.0961^{ns}/Yes	0.8822/0.1110^{ns}/Yes	0.9608/0.7063^{ns}/Yes	0.9104/0.2841^{ns}/Yes
Coliforms log_{10}(CFU+1)/WS (n=15)	0.9408/0.3280^{ns}/Yes	0.8242/0.0076**/No	0.8647/0.0282*/No	0.8413/0.0170*/No
Coliforms log_{10}(CFU+1)/V/cow (n=12)	0.8989/0.1086^{ns}/Yes	0.8975/0.1724^{ns}/Yes	0.9709/0.8709^{ns}/Yes	0.8500/0.0581^{ns}/Yes

Notes: Values are presented as Shapiro–Wilk W statistic, p-value, and normality decision (Yes/No). Normal distribution was assumed at $p > 0.05$. RH—relative humidity; PM – particle matters; TVOCs – Total Volatile Organic Compounds; BM – Bedding Moisture; WS – Wind Speed; V/cow – volume per cow.

Table S3. Results of the Shapiro–Wilk test for normality of log_{10}-transformed Molds in the studied farms

Variable	Farm 1 (W / p/ Yes-No)	Farm 2 (W / p/ Yes-No)	Farm 3 (W / p/ Yes-No)	Farm 4 (W / p/ Yes-No)
Molds log_{10}(CFU+1) (n=15)	0.9103/0.1011^{ns}/Yes	0.8487/0.0166*/No	0.8894/0.054^{ns}/Yes	0.9481/0.495^{ns}/Yes
Molds log_{10}(CFU+1)/RH (n=15)	0.9343/0.2848^{ns}/Yes	0.8285/0.0115*/No	0.9291/0.2646^{ns}/Yes	0.9646/0.7975^{ns}/Yes
Molds log_{10}(CFU+1)/PM1 (n=15)	0.8741/0.0314*/No	0.7416/0.0010**/No	0.8633/0.0269*/No	0.7387/0.0010***/No
Molds log_{10}(CFU+1)/PM2.5 (n=15)	0.9347/0.2895^{ns}/Yes	0.7417/0.0010**/No	0.8847/0.0559^{ns}/Yes	0.9096/0.1556^{ns}/Yes
Molds log_{10}(CFU+1)/PM10 (n=15)	0.9079/0.1075^{ns}/Yes	0.7407/0.0010**/No	0.8931/0.0746^{ns}/Yes	0.9096/0.1556^{ns}/Yes
Molds log_{10}(CFU+1)/TVOCs (n=15)	0.9618/0.6946^{ns}/Yes	0.9648/0.8011^{ns}/Yes	0.9591/0.6767^{ns}/Yes	0.9389/0.4040^{ns}/Yes
Molds log_{10}(CFU+1)/Hbed (n=12)	0.9517/0.6249^{ns}/Yes	0.7190/0.0008***/No	0.9407/0.4664^{ns}/Yes	0.9449/0.5802^{ns}/Yes
Molds log_{10}(CFU+1)/WS (n=15)	0.8712/0.0283*/No	0.8547/0.0257*/No	0.8647/0.0282*/No	0.8338/0.0135*/No
Molds log_{10}(CFU+1)/V/cow (n=12)	0.9183/0.2382^{ns}/Yes	0.8326/0.0252*/No	0.9594/0.7440^{ns}/Yes	0.8420/0.0335*/No

Notes: Values are presented as Shapiro–Wilk W statistic, p-value, and normality decision (Yes/No). Normal distribution was assumed at $p > 0.05$. RH—relative humidity; PM – particle matters; TVOCs – Total Volatile Organic Compounds; BM – Bedding Moisture; WS – Wind Speed; V/cow – volume per cow.

Table S4. Results of the Kruskal–Wallis test for the studied variables

Variables	Kruskal-Wallis H	df	Asymp. Sig. P – values
TBC, log_{10}(CFU+1)	5.134	3	0.162
TBC log_{10}(CFU+1)/RH	5.955	3	0.114
**TBC log_{10}(CFU+1)/PM1**	**11.37**	**3**	**0.01**
**TBC log_{10}(CFU+1)/PM2,5**	**11.46**	**3**	**0.009**
**TBC log_{10}(CFU+1)/PM10**	**11.51**	**3**	**0.009**
TBC log_{10}(CFU+1)/TVOCs	8.669	3	0.034
**TBC log_{10}(CFU+1)/Bedding Moisture**	**29.85**	**3**	**<0.001**
TBC log_{10}(CFU+1)/Wind Speed	10.89	3	0.012
**TBC log_{10}(CFU+1)/Volume per cow**	**42.32**	**3**	**<0.001**
Coliforms log_{10}(CFU+1)	9.504	3	0.023
**Coliforms log_{10}(CFU+1)/RH**	**13.05**	**3**	**0.005**
**Coliforms log_{10}(CFU+1)/PM1**	**16.24**	**3**	**0.001**
**Coliforms log_{10}(CFU+1)/PM2,5**	**17.29**	**3**	**<0.001**
**Coliforms log_{10}(CFU+1)/PM10**	**16.62**	**3**	**<0.001**
**Coliforms log_{10}(CFU+1)/TVOCs**	**12.32**	**3**	**0.006**
**Coliforms log_{10}(CFU+1)/Bedding Moisture**	**15.42**	**3**	**0.001**
**Coliforms log_{10}(CFU+1)/Wind Speed**	**18.39**	**3**	**<0.001**
**Coliforms log_{10}(CFU+1)/Volume per cow**	**38.54**	**3**	**<0.001**
Molds log_{10}(CFU+1)	1.805	3	0.614
**Molds log_{10}(CFU+1)/RH**	**30.17**	**3**	**<0.001**
Molds log_{10}(CFU+1)/PM1	13.98	3	0.003
**Molds log_{10}(CFU+1)/PM2,5**	**19.65**	**3**	**<0.001**
**Mouds log_{10}(CFU+1)/PM10**	**18.27**	**3**	**<0.001**
**Molds log_{10}(CFU+1)/TVOCs**	**28.11**	**3**	**<0.001**
**Molds log_{10}(CFU+1)/Bedding Moisture**	**36.52**	**3**	**<0.001**
Molds log_{10}(CFU+1)/Wind Speed	3.512	3	0.319
**Molds log_{10}(CFU+1)/Volume per cow**	**40.04**	**3**	**<0.001**
